# Colloid of the Brain

**Published:** 1869-04-01

**Authors:** 


### *Colloid of the Brain.*

M. Magnan presented, recently, to the Biological Society of Paris, preparations of colloid substance observed upon the surface of the brain of a patient who had yielded gradually to the progress of general paralysis. The colloid substance was located in the gray substance of the convolutions of the frontal and speroidal lobes. It was opaline in color, and depressed the cerebral substance. It appeared in the form of small islets. Examined with the microscope, there were observed in it concentric disks of a brilliant opaline matter, studded with shining nuclei, and in the middle of the central disk, a capillary vessel with thickened walls was perceived. The nervous substance itself was altered in the same manner as the connective tissue; that is to say, it was shining like the nuclei. By comparative examinations of several preparations M. Magnan has been led to think that the histological changes commenced in the nuclei, and invaded secondarily the nervous cells.

Treated with tincture of iodine and ether, these preparations preserved their primitive aspect. The alterations were, therefore, due neither to fat nor to starchy matter. —*Ibid.*

M. Dumontpallier called the attention of the Biological Society to the frequency of permanent congestions of the ears of the insane, and especially of the subjects of general paralysis. No physician can traverse the corriders or dormitories of insane asylums without being struck with the appearance of this congestion of the ears, which has, moreover, a direct relation to congestion of the cerebral membranes.—*Ibid.*

